# Protocol for monitoring of neutralizing antibodies to various receptor-binding domains using the tANCHOR system

**DOI:** 10.1016/j.xpro.2025.103926

**Published:** 2025-07-02

**Authors:** Daniel Ivanusic, Norbert Bannert

**Affiliations:** 1 Sexually Transmitted Bacterial Pathogens and HIV (FG18), Robert Koch Institute, 13353 Berlin, Germany

**Keywords:** Cell Membrane, Cell-based Assays, High-Throughput Screening, Immunology, Microbiology, Antibody, Protein expression and purification

## Abstract

Neutralizing antibody analysis against SARS-CoV-2 variants requires assays that rapidly adapt to protein mutations. Here, we present a protocol for monitoring neutralizing antibodies to various receptor-binding domains using the tANCHOR system. We describe steps for displaying variant receptor-binding domains on HeLa cells and producing tagged soluble angiotensin-converting enzyme 2 (ACE2). We then detail the procedures for establishing a cell-based ELISA to measure serum neutralization efficiency, using ACE2 competition as a readout.

For complete details on the use and execution of this protocol, please refer to Ivanusic et al.^1^

## Highlights

• Cloning SARS-CoV-2 RBD into tANCHOR for surface display on HeLa cells
• Establishing stable HEK293T cells secreting soluble ACE2 protein
• Preparing 96-well plates with RBD-displaying HeLa cells
• Guidance on ACE2 competition cell-based ELISA to assess neutralizing activity

## Abstract (editor highlights)

Publisher’s note: Undertaking any experimental protocol requires adherence to local institutional guidelines for laboratory safety and ethics.

## Graphical abstract

**Figure:**
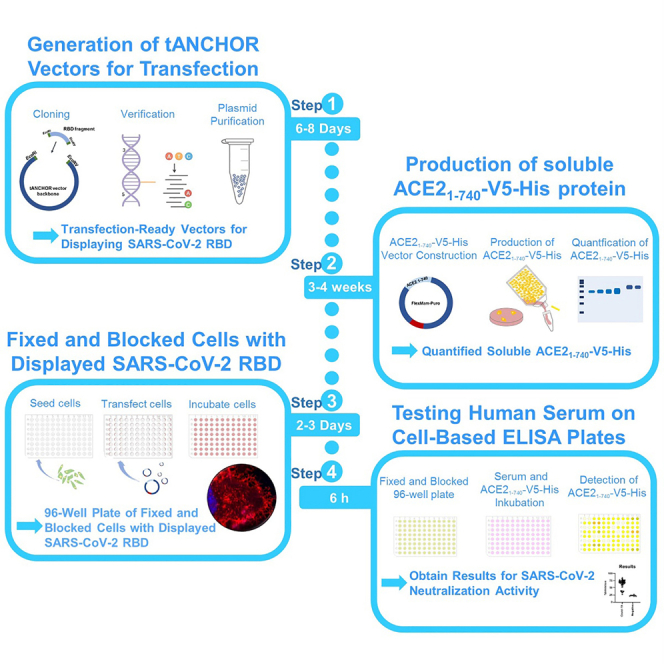

## Before you begin

SARS-CoV-2, the virus responsible for the COVID-19 pandemic, has evolved into multiple variants with distinct transmissibility and immune escape characteristics.^2^ Monitoring of neutralizing antibodies (nAbs) against these variants is essential for guiding vaccine development and public health strategies. Here, we describe a detailed and adaptable protocol for monitoring neutralizing antibodies directed against the receptor-binding domain (RBD) of SARS-CoV-2 variants using a cell-based assay. This protocol outlines the preparation of necessary components, which requires approximately 4 to 5 weeks, followed by a cell-based neutralization assay using human serum samples to test for neutralizing antibodies, which can be completed within 4 to 5 days. Importantly, the prepared assay components are stable for storage and can be reused for subsequent analyses, enabling laboratories to efficiently perform high-throughput neutralization testing for emerging SARS-CoV-2 variants. In addition to its utility for antibody screening, this protocol can also be applied in gain-of-function research to assess the binding affinity of angiotensin-converting enzyme 2 (ACE2) to different RBD variants. In this case, antibodies are not applied and only the prepared ACE2_1-740_-V5-His protein is used in the protocol. By testing hypothetical or emerging RBD sequences, it is possible to identify variants with enhanced ACE2 binding affinity—even before they appear in the population. This gain in binding strength, coupled with a loss of neutralizing activity by existing antibodies, can serve as an early warning signal for potential immune escape and increased transmissibility. In summary, this cell-based assay protocol for measuring SARS-CoV-2 neutralizing antibodies provides a forecasting capability, supporting preparedness for future variants with increased viral fitness and advanced immune escape potential.

### Institutional permissions

Permission from the institutional ethics commission is needed before using human material. We would like you to be aware of which local permits are necessary for the use of human material in your laboratory. In Germany where we carried out the experiments, we obtained this permission according to ethical guidelines of the 1975 Declaration of Helsinki and it was approved by the institutional ethics committee of the University of Freiburg (EK 153/20). Participants provided written informed permission, and the research was carried out in compliance with federal guidelines and the rules of the local ethics committee (F-2020-09-03-160428, No. 322/20, and No 20-1271_1). Participants received no payment.

## Key resources table

**Table:** 

REAGENT or RESOURCE	SOURCE	IDENTIFIER
**Antibodies**		

Mouse anti-V5-HRP, dilution 1:8,000	Invitrogen by Thermo Fisher Scientific	Cat# 46–0708, R961-25
Rabbit anti-ACE2, dilution 1:5,000	Invitrogen by Thermo Fisher Scientific	Cat# MA-32307

**Bacterial and virus strains**		

Chemocompetent *E. coli* DH5α	New England Biolabs	Cat# C2987H

**Biological samples**		

Human serum, obtained under ethical approval EK 153/20	University Medical Center, Freiburg	N/A

**Chemicals, peptides, and recombinant proteins**		

*Eco*RI-HF	New England Biolabs	Cat# R3101
*Eco*RV-HF	New England Biolabs	Cat# R3195
*Pme*I	New England Biolabs	Cat# R0560
*Nhe*I-HF	New England Biolabs	Cat# R3131
T4 DNA ligase	New England Biolabs	Cat# M0202
Paraformaldehyde (PFA)	Carl Roth	Cat# 0335.1
Bovine serum albumin fraction V (BSA)	Carl Roth	Cat# T844.2
Chicken egg albumin (CEA)	Sigma-Aldrich	Cat# A5253
Normal goat serum (NGS)	Biowest	Cat# S2000-500
DMEM high glucose medium	Robert Koch-Institute, in-house preparation	Order No# 8
1× Phosphate-buffered saline **(**PBS)	Robert Koch-Institute, in-house made	Order No# 27
Terrific broth (TB) medium	Robert Koch-Institute, in-house made	N/A
Fetal bovine serum (FBS)	Gibco	Cat# 11573397
Penicillin-Streptomycin 100 ×	Gibco	Cat# 15140122
Puromycin	Carl Roth	Cat# 0240.4
Ampicillin sodium salt	Carl Roth	Cat# K029.3
L-glutamine (200 mM)	Gibco	Cat# 25030081
Ethidium bromide 10 mg/mL	Bio-Rad	Cat# 161-0433
Agarose	Thermo Scientific	Cat# 16500-500
Trypsin-EDTA	Gibco	Cat# 25200056
Metafectene	Biontex	Cat# T020
Tween 20	Sigma-Aldrich	Cat# 93773
Methanol	Carl Roth	Cat# 4627.2
Acetic acid 100%	Carl Roth	Cat# 3738.5
TMB (3,3′,5,5′-tetramethylbenzidine) substrate 11× and TMB buffer	Bio-Rad	Cat# TMB7BR
2 M H_2_SO_4_ (TMB stop solution)	Bio-Rad	Cat# STP1BR
NaCl	Carl Roth	Cat# 3957
KCl	Carl Roth	Cat# HN02
Dimethyl sulfoxide (DMSO)	Carl Roth	Cat# A994.1
Na_2_HPO_4_	Carl Roth	Cat# P030
Glycerol	Carl Roth	Cat# 3783.5
KH_2_PO_4_	Carl Roth	Cat# P018
Coomassie Brilliant Blue R-250	Carl Roth	Cat# 3862.2
NaOH 2M	Carl Roth	Cat# T135.1
Agar-Agar, bacteriological	Carl Roth	Cat# 2266.2
PageRuler prestained protein ladder, 10 to 180 kDa	Thermo Scientific	Cat# 26616
2× Laemmli buffer	Sigma-Aldrich	Cat# S340-1VL
Nanobody/VHH V5-Trap magnetic agarose	ChromoTek	Cat# V5tma
Hoechst 33342 stain	Immunochemistry	Cat# 639
Pierce BSA standard 2 mg/mL	Thermo Scientific	Cat# 23209

**Critical commercial assays**		

QIAprep Spin Miniprep Kit, see manual on www.qiagen.com in the section DNA & RNA → Purification → DNA→ Plasmid DNA	QIAGEN	Cat# 27106
QIAGEN Plasmid Maxi Kit, see manual on www.qiagen.com in the section DNA & RNA → Purification → DNA→ Plasmid DNA	QIAGEN	Cat# 12163

**Experimental models: Cell lines**		

HeLa	ATCC	CCL-2
HEK293T	ATCC	CRL-3216

**Oligonucleotides**		

Seq.1_for 5′-→ 3′: AACGAGGTCCGCTGCCTG	Integrated DNA Technologies	N/A

**Recombinant DNA**		

ptANCHOR-CD82-V5-mCherry	Ivanusic et al.^1^	ATG:biosynthetics
FlexMam-Puro	Ivanusic et al.^1^	ATG:biosynthetics

**Software and algorithms**		

GraphPad Prism 9.2.0	Graphpad Software Inc	www.graphpad.com
Microsoft Office Professional 2019	Microsoft	www.microsoft.com
Fiji	Open-source platform	http://fiji.sc
SnapGene	Dotmatics	www.snapgene.com

**Other**		

96-well tissue culture testplate 96F	TPP	Cat# 92096
6-well tissue culture plate	TPP	Cat# 92006
Tissue culture tube 15 mL	TPP	Cat# 91015
Tissue culture tube 50 mL	TPP	Cat# 91050
Tissue cell culture flask 300 cm^2^	TPP	Cat# 90301
Tissue cell culture flask 150 cm^2^	TPP	Cat# 90151
Tissue cell culture flask 75 cm^2^	TPP	Cat# 90076
Tissue cell culture flask 25 cm^2^	TPP	Cat# 90026
Microplate reader Infinite 200	Tecan	www.tecan.com
Microplate washer BioTec 405	Agilent Technologies	www.agilent.com
Microplate seal film ROTILABO	Carl Roth	Cat# EN76.1
Improved Neubauer cell chamber	Brand	www.brand.de
Fluorescence microscope AxioObserver 20× objective, Axiocam 503 mono, filter cube for red dyes	Zeiss	www.zeiss.com
Confocal laser scanning microscope LSM 780	Zeiss	www.zeiss.com
NEX5 digital camera with a SONY macro E3.5/30 objective	Sony, Japan	www.sony.com
Mr. Frosty cell freezing container	Thermo Scientific	Cat# 15-350-50
Cryovial 2 mL	Greiner Bio-One	Cat# 126277
4%–15% Mini-Protean TGX precast gel	Bio-Rad	Cat# 4561083
1.5 ml reaction tubes	Carl Roth	Cat# 1KP0.1
PCR reaction tubes ROTILABO	Carl Roth	Cat# CEN5.1
ROTILABO ELISA seal film	Carl Roth	Cat# EN76.1

## Materials and equipment

**Table:** TB medium

Reagent	Final concentration	Amount
Tryptone	12 g/L	12 g
Yeast extract	24 g/L	24 g
Glycerol	0.4% v/v	4 mL
KH_2_PO_4_	0.017 M	2.3 g
K_2_HPO_4_	0.072 M	12.5 g
Deionized water	N/A	Up to 1 L
Total volume		1 L, adjust to pH 7.2, autoclave

Autoclave and store at 4°C, stable for three months.

**Table:** DMEM/FBS

Reagent	Final concentration	Amount
FBS	10%	100 mL
Penicillin (10,000 U/mL) -Streptomycin (10,000 μg/mL)	100 U/mL and 100 μg/mL	10 mL
L-Glutamine (200 mM)	2 mM	10 mL
DMEM	N/A	Up to 1 L
Total volume		1 L

Store at 4°C, use within 14 days.

**Table:** DMEM/FBS/puromycin 2 μg/mL

Reagent	Final concentration	Amount
FBS	10%	100 mL
Puromycin 10 mg/mL (sterile filtered, 0.22 μm)	2 μg/mL	200 μL
Penicillin (10,000 U/mL) -Streptomycin (10,000 μg/mL)	100 U/mL and 100 μg/mL	10 mL
L-Glutamine (200 mM)	2 mM	10 mL
DMEM	N/A	Up to 1 L
Total volume		1 L

Store at 4°C, use within 14 days.

**Table:** DMEM/FBS/puromycin 5 μg/mL

Reagent	Final concentration	Amount
FBS	10%	100 mL
Puromycin 10 mg/mL (sterile filtered, 0.22 μm)	5 μg/mL	500 μL
Penicillin (10,000 U/mL) -Streptomycin (10,000 μg/mL)	100 U/mL and 100 μg/mL	10 mL
L-Glutamine (200 mM)	2 mM	10 mL
DMEM	N/A	Up to 1 L
Total volume		1 L

Store at 4°C, use within 14 days.

**Table:** 1× Phosphate-buffered saline **(**PBS)

Reagent	Final concentration	Amount
NaCl	137 mM	8 g
KCl	2.7 mM	0.2 g
Na_2_HPO_4_	10 mM	1.42 g
KH_2_PO_4_	1.47	0.24
Deionized water	N/A	Up to 1 L
Total volume		1 L, adjust to pH 7.4

Autoclave and store at RT, stable for six months.

**Table:** Cell fixation buffer PFA

Reagent	Final concentration	Amount
Paraformaldehyde (PFA)	2%	20 g
1× PBS	N/A	Up to 1 L
Total volume		1 L

Store at 4°C, stable for one month.

**Table:** Blocking buffer

Reagent	Final concentration	Amount
Chicken egg albumin	2%	20 g
Bovine Albumin Fraction V	3%	30 g
Normal goat serum	10 %	100 mL
1× PBS	N/A	Up to 1 L
Total volume		1 L

Store at 4°C, stable for one week.

**Table:** Wash buffer

Reagent	Final concentration	Amount
Tween 20	0.05%	0.5 mL
1× PBS	N/A	Up to 1 L
Total volume		1 L

Store at 4°C, stable for one month.

**Table:** Coomassie staining solution

Reagent	Final concentration	Amount
Brilliant Blue R-250	0.1%	1 g
Methanol	50%	400 mL
Acetic acid 100%	10%	100 mL
Deionized water	N/A	Up to 1 L
Total volume		1 L

Storage at RT protected from light, stable for 6 months.

**Table:** Coomassie destain solution

Reagent	Final concentration	Amount
Methanol	40%	400 mL
Acetic acid 100%	10%	100 mL
Deionized water	N/A	Up to 1 L
Total volume		1 L

Store at RT, stable for one year.

## Step-by-step method details

### Cloning and preparation of tANCHOR-RBD vectors for cell surface expression

**Timing: 6–8 days**

**Figure 1:**
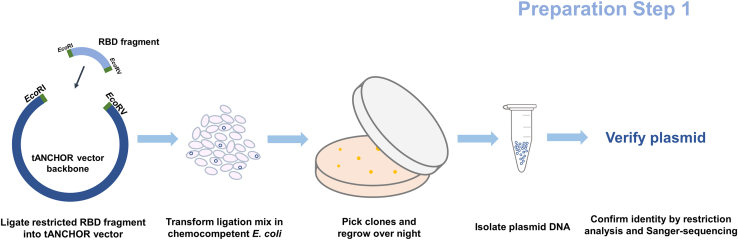
Overview of the workflow for cloning RBD coding DNA fragments into the tANCHOR vector system The RBD coding fragment is inserted between the restriction sites *Eco*RI and *Eco*RV. The ligation mix is plated on agar containing 100 μg/mL ampicillin. Colonies are propagated in TB medium with 100 μg/mL ampicillin, and plasmid DNA is isolated for sequence confirmation.

Before measuring neutralizing antibodies, it is essential to prepare expression constructs using standard cloning methods. This section describes the steps for generating expression vectors designed to display the receptor-binding domain (RBD) on the cell surface. For common pitfalls during cloning, please refer to Matsumura.^3^ Figure 1 provides an overview of the main steps of the RBD cloning.

1. Collect coding sequence information of the SARS-CoV-2 RBD to be tested. The typical RBD region contains the amino acids (aa) 318-543 within the SARS-CoV-2 spike protein.^1^^,^^4^^,^^5^

**Figure 2:**
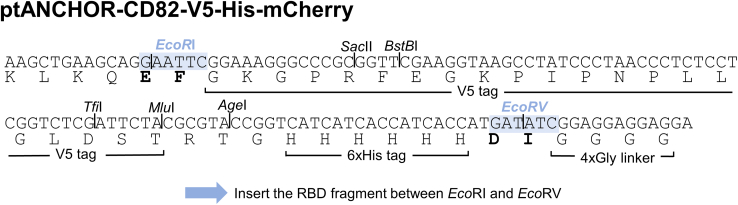
In-frame cloning of the RBD into the tANCHOR vector system The RBD or protein of interest is inserted between the restriction sites *Eco*RI and *Eco*RV. The coding region has to be in frame with the 4× glycine linker.

***Note:*** The RBD sequence has to be in frame with the 4× glycine (Gly) linker. Use cloning software such as SnapGene to design the cloning strategy (Figure 2).

2. Order the RBD sequence in a version that is codon optimized for expression in human cells flanked by *Eco*RI (GAATTC) and *Eco*RV (GATATC) sites for cloning into the tANCHOR vector ptANCHOR-CD82-V5-His-mCherry.

***Note:*** Alternatively, the RBD sequence can be PCR-amplified from a suitable template using forward and reverse primers containing the *Eco*RI and *Eco*RV restriction sites. Make sure the reading frame is correct and that the primers introduce suitable overhangs for efficient cloning.

3. Cut the gene synthesis RBD fragment and target vector with the mentioned restriction enzymes.
  a. Adjust the plasmid containing codon optimized synthetic RBD sequence to a concentration of 0.5 μg/μL.
  b. then proceed with the following digestion setup:
    i. Add 1 μg (2 μL) to 41 μL of sterile deionized (s.d.) water in a PCR reaction tube (Carl Roth).
    ii. Add then 5 μL 10× rCutSmart or r2.1 buffer (NEB).
    iii. Add 1 μL each of the restriction enzymes *Eco*RI-HF (NEB) and *Eco*RV-HF (NEB) for a double digest process.

***Note:*** The final volume should always be 50 μL. Adjust the amount of s.d. water accordingly if the DNA is dissolved in a different concentration or volume.

4. In the same way digest 1.5 μg of the tANCHOR vector ptANCHOR-CD82-V5-His-mCherry.
5. Incubate the restriction mix for 4 h at 37°C.
6. Add loading buffer and separate DNA by a 1% agarose gel containing 0.2 μg/mL ethidium bromide. Cut the bands with the vector backbone and the RBD fragment out of the gel. An observable band of approximately 700 base pairs (bp) should appear.
7. Purify DNA by using Gel purification kit (Qiagen) and eluate DNA in 30 μL of s.d. water.
8. Measure DNA concentration using a suitable device like the NanoVue from GE Healthcare.
9. Ligate tANCHOR backbone RBD fragment at a molar ratio of ∼1:3 (vector:insert).
  a. Add 100 ng of the tANCHOR backbone and 50 ng of the RBD fragment into a PCR reaction tube (Carl Roth).
  b. Mix and add 2.5 μL T4 DNA Ligase Reaction Buffer 10× (NEB).
  c. Add s.d. water to bring the total volume to 24 μL, then mix gently.
  d. Add 1 μL T4 DNA ligase (NEB), then mix gently.

***Note:*** You should also set up a ligation mixture without the RBD insert as a control.

10. Incubate ligation mixture first for 1 h at room temperature (RT, approximately 20°C–25°C), then for 16–18 h at 16°C using a PCR cycler.
11. Heat inactivate for 10 min at 65°C.
12. Transform ligation mix to DH5α chemically competent *E. coli* cells with a transformation efficiency of 1 - 3 x 10^9^ cfu/μg pUC19 DNA (NEB).
  a. Thaw DH5α chemically competent *E. coli* cells, stored at −80°C, on ice for 10 min.
  b. Add 10 μL of the ligation mixture to 50 μL of DH5α chemically competent cells.
  c. Incubate the tube on ice for 10 min. Do not vortex; instead, gently flick the tube 4–5 times to mix.
  d. Incubate the mixture on ice for 30 min.
  e. Place the tube in a ThermoMixer (Eppendorf) or an equivalent device without mixing, and perform a heat shock at exactly 42°C for 30 s.
  f. Place tube on ice for 5 min.
  g. Add 950 μL of SOC medium (NEB) equilibrated to RT.
  h. Incubate the tube at 37°C for 60 min with vigorous shaking at 250 rpm.
  i. Spread 100 μL and the remaining ∼900 μL of the transformation mixture onto pre-warmed LB agar plates containing 100 μg/mL ampicillin.
  j. Incubate plates for 16–18 h at 37°C.

***Note:*** Pick colonies only if there are at least three times as many colonies on the plate containing the ligation mix with both vector and insert compared to the control plate containing the ligation mix with vector but without the RBD insert. Otherwise, optimize the restriction of the tANCHOR vector and use a heat-labile version of calf intestinal alkaline phosphatase (CIP), such as Quick CIP (NEB), to increase the number of positive clones.

13. Pick 3–5 colonies and regrow transformed *E. coli* for 16–18 h in 10 mL TB medium at 37°C containing 100 μg/mL ampicillin.
14. Isolate plasmid DNA using the Qiagen plasmid isolation kit mini, eluate plasmids from the column using 50 μL of s.d. water.
15. Use the remaining 16–18 h *E. coli* culture to prepare glycerol stock for long term storage.
  a. Add 500 μL of *E. coli* culture to 500 μL of an autoclaved 50% (v/v) glycerol/water solution (1:1) in a sterile 2 mL cryovial (e.g., Greiner Bio-One).
  b. Gently mix the vial until no mixing streaks are visible and the solution appears homogeneous.
  c. Freeze the tube immediately at −80°C.
16. Verify Cloning of the RBD Insert.
  a. Check DNA insert of the RBD by *Eco*RI/*Eco*RV restriction analysis of the isolated plasmids. Presence of the RBD insert should appear at ∼700 bp.
  b. Sequence the tANCHOR vector for sequence confirmation using the primer Seq.1_for for Sanger-sequencing as described in Bernauer et al.^4^^,^^6^
17. Purification of Plasmid DNA for transfection.
  a. Inoculate 20 μL of glycerol stock containing the confirmed tANCHOR vector with RBD insert into 250 mL of pre-warmed (37°C) TB medium supplemented with 100 μg/mL ampicillin.
  b. Grow the culture at 37°C with shaking for ∼16–18 h.
  c. Plasmid DNA was isolated using the Qiagen Maxi Kit, following manufacturer’s instructions for standard laboratory procedure.^4^
  d. Elute plasmid DNA in s.d. water and adjust the concentration to 500 ng/μL.
  e. Measure plasmid DNA concentration using a NanoVue (GE healthcare) or equivalent spectrophotometer.
  f. Aliquot the purified plasmid DNA and store at −20°C until use.

***Note:*** Dilute the plasmid DNA to a concentration of 500 ng/mL with s.d. water. This helps to minimize pipette errors compared to using highly concentrated plasmid DNA when setting up the transfection mix.

### Preparation of supernatants containing soluble ACE2_1-740_-V5-His protein

**Timing: 3–4 weeks**

**Figure 3:**
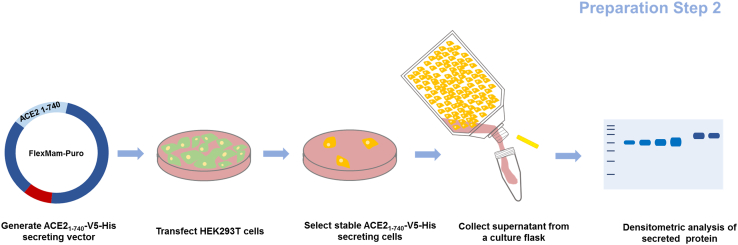
Workflow for the production of supernatant containing secreted ACE2_1-740_-V5-His protein The stable HEK293T cell line secretes soluble ACE2_1-740_-V5-His protein, which is used as one of the components to evaluate antibody competition in the binding of ACE2 to the RBD.

The next step involves cloning a secretion vector encoding soluble human angiotensin-converting enzyme 2 (ACE2) protein fused to V5 and 6× histidine tags for detection and isolation. This vector is used to generate a stable HEK293T cell line that continuously secretes the tagged ACE2 protein. Figure 3 illustrates the workflow for cloning the secretion construct, establishing the stable cell line, and performing preliminary quantification of the secreted ACE2_1-740_-V5-His protein, which is a key reagent for the SARS-CoV-2 cell-based neutralization assay.

**Figure 5:**
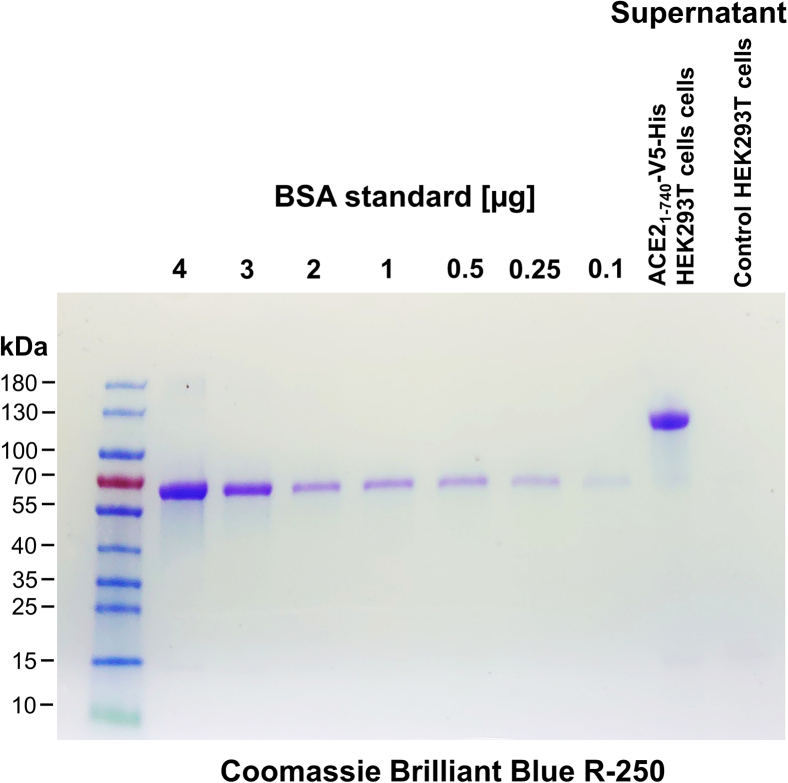
Quantification of secreted ACE2_1-740_-V5-His protein The protein was isolated from the supernatant of a stably transfected HEK293T cell line and separated by SDS-PAGE (4%–15% Mini-PROTEAN TGX gel, Bio-Rad) and stained with Coomassie Brilliant Blue R-250. For quantification, signal intensity is compared to a BSA standard. If the stained ACE2_1-740_-V5-His band from 500 μL supernatant aligns with the 4 μg BSA band, the sample volume should be adjusted accordingly. Protein molecular weights were estimated using the PageRuler Prestained Protein Ladder (10–180 kDa).
  o. Use the software ImageJ/Fiji to generate a standard curve using BSA bands. To do this, select the "Region" option and then choose "Analyze Gels → Plot Lanes" in the software. Calculate from densitometric data the amount of pACE2_1-740_-V5-His protein.
  p. Calculate the amount of secreted protein using data from the BSA standard curve. Typically, 7 μg/mL can be obtained in the supernatant from a 300 cm^2^ cell culture flask cultivated over 3 days with 50 mL of medium.
    ***Note:*** Secreted ACE2_1-740_-V5 protein can be additionally characterized by Western blot analysis using antibodies raised against ACE2 (Thermo Fisher Scientific).

18. Insert the coding sequence for human ACE2 containing the aa 1-740 in the FlexMam-Puro vector system (ATG:biosynthetics).
  a. Perform the cloning steps as described in the cloning section for the RBD insert. Order the gene fragment flanked by *Nhe*I (GCTAGC) and *Pme*I (GTTTAAAC) restriction sites for the coding region of the human ACE2 protein (amino acids 1-740) that will secrete a soluble protein form.^1^^,^^4^^,^^7^
  b. Isolate plasmid DNA from *E. coli* for cell transfection. Adjust DNA concentration to 500 ng/μL.
  c. Confirm the identity of the ACE2_1–740_-V5-His fragment by restriction enzyme analysis and Sanger sequencing.
  d. Isolate plasmid DNA for transfection for HEK293T cells using Qiagen maxi kit and adjust DNA concentration to 500 ng/μL.
19. Generation of stable HEK293T cells secreting the protein pACE2_1-740_-V5.
  a. Seed 3 × 10^5^ HEK293T cells (DMEM with FBS) per well in a 6-well cell culture plate (TPP).
  b. Incubate in a cell culture incubator at 37°C, 5% CO_2_, relative humidity 90%–95% until cells reach 70%–80% confluency (typically after 16–18 h).
  c. Transfect cells using Metafectene.
    i. Prepare two reaction tubes, each containing 100 μL of DMEM without fetal calf serum (FBS) and without antibiotics.
    ii. Dilute 4 μg plasmid DNA of the vector pACE2_1-740_-V5-His in one tube and 8 μL of Metafectene in the other tube. Mix through one-time pipetting and mix both together.
    iii. incubate transfection mix for 20 min at RT.
  d. Add transfection mix dropwise to one 6-well and incubate for 24 h in the cell culture incubator at 37°C, 5% CO_2_, relative humidity 90%–95%.
    ***Note:*** Cells should not be too dense, as this can reduce transfection efficiency and lead to increased cell death. Ensure optimal cell density for the best results.
  e. Remove the supernatant and replace the medium by adding 2 mL of pre-warmed (37°C) DMEM with FBS and 5 μg/mL puromycin (Carl Roth) and incubate for 3 days in a cell culture incubator at 37°C, 5% CO_2_, relative humidity 90%–95%.
  f. Remove the supernatant and dead cells.
  g. Add 2 mL of pre-warmed (37°C) DMEM with FBS and 2 μg/mL puromycin to each well.
    ***Note:*** When removing the supernatant, be careful not to detach any live cells. After 7 days, the 6-well plate should display visible small cell clusters (see Figure 4). To obtain more viable cells for further propagation into a larger cell culture flask, we recommend using cells from the three transfected wells.
    **Figure 4:**
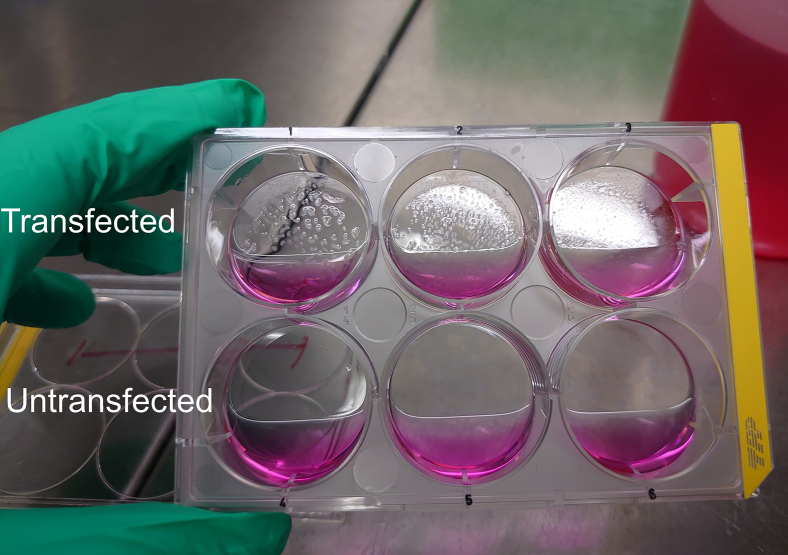
Appearance of transfected cells after puromycin selection Images show typical cell clusters observed one week post-selection (top row). Untransfected control cells (bottom row) are completely eliminated by puromycin and washed out during cell culture medium changes.
  h. Incubate cells at 37°C, 5% CO_2_, relative humidity 90%–95% for several days (typically 6–8 days) until the 6-well plate contains enough cells to passage in a 25 cm^2^ growth area cell culture flask (TPP).
    i. Wash cells once with 1 mL 1× PBS.
    ii. Add 100–200 μL pre-warmed (37°C) trypsin-EDTA (Sigma-Aldrich).
    iii. When cells are starting detaching, add immediately 2 mL pre-warmed (37°C) DMEM containing FBS.
    iv. Transfer cells into a 15 mL cell culture tube (TPP) and centrifuge (900 rpm, 3 min, RT), remove supernatant.
    v. Wash once with 5 mL of 1× PBS, and centrifuge (900 rpm, 3 min, RT) again.
    vi. Add 10 mL of pre-warmed (37°C) DMEM containing FBS and 2 μg/mL puromycin.
    vii. Seed cells into 25 cm^2^ cell culture flask.
    viii. Incubate at 37°C, 5% CO_2_, relative humidity 90%–95% until cells reach a high level of confluency.
  i. Passage cells to a 75 cm^2^ cell culture flask.
    i. Wash cells in the cell culture flask once with 10 mL of 1× PBS.
    ii. Trypsinize cells by adding 1 mL of pre-warmed (37°C) trypsin-EDTA.
    iii. Add 10 mL of pre-warmed (37°C) DMEM containing FBS and 2 μg/mL puromycin.
    iv. Transfer cells into a 15 mL cell culture tube and centrifuge (900 rpm, 3 min, RT).
    v. Wash cells once with 1× PBS (900 rpm, 3 min, RT).
  j. Resuspend the cells in 10 mL of pre-warmed (37°C) DMEM containing FBS and 2 μg/mL puromycin. Transfer half of the cell suspension (5 mL) into a 75 cm^2^ cell culture flask and add 10 mL of pre-warmed (37°C) DMEM containing FBS and 2 μg/mL puromycin.
  k. Store remaining cells in liquid nitrogen as backup.
    i. Centrifuge residual cells at 900 rpm, 3 min, RT and remove supernatant.
    ii. Add 3 mL FBS containing 10% DMSO to cells and mix gently.
    iii. Add 1 mL in each labeled cryovial.
    iv. Decrease the temperature approximately 1°C per minute in a −80°C freezer by using a freezing apparatus (Mr. Frosty, Thermo Fisher Scientific) for controlling the freezing rate.
    v. After 24 h store cryovials in liquid nitrogen.
  l. Passage cells to a 150 cm^2^ cell culture flask.
    i. Wash cells in the cell culture flask once with 15 mL of 1× PBS.
    ii. Trypsinize cells by adding 3 mL of pre-warmed (37°C) trypsin-EDTA.
    iii. Add 20 mL of pre-warmed (37°C) DMEM containing FBS and 2 μg/mL puromycin.
    iv. Transfer cells into a 50 mL cell culture tube and centrifuge (900 rpm, 3 min, RT).
    v. Wash cells once with 25 mL 1× PBS (900 rpm, 3 min, RT).
    vi. Resuspend cells in 25 mL of pre-warmed (37°C) DMEM containing FBS and 2 μg/mL puromycin.
    vii. Place cell suspension into a 150 cm^2^ cell culture flask.
  m. Perform cell expansion steps until there are enough cells to seed a 300 cm^2^ cell culture flask to achieve 70%–80% confluency for 16–18 h.
  n. Collect supernatant containing ACE2_1-740_-V5-His protein.
    i. Remove supernatant from the cells at 70%–80% confluency cultivated in a 300 cm^2^ cell culture flask.
    ii. Add 50 mL DMEM with 2% FBS and 2 μg/mL puromycin.
    iii. Incubate cells for 3 days at 37°C, 5% CO_2_, relative humidity 90%–95%.
    iv. Collect the supernatant and centrifuge it (4,000 rpm, 10 min, 4°C) to remove cell debris.
  o. Adjust the pH of the solution to 7.2 using 2 M NaOH while stirring continuously.
  p. Freeze the supernatant aliquots at −80°C for long-term storage.
    ***Note:*** The stable HEK293T cell line secreting ACE2_1-740_-V5-His protein should be maintained in DMEM supplemented with 2 μg/mL puromycin. In the absence of selective pressure, some cells may lose or transcriptionally silence the integrated construct. This selective pressure maintains a stable, high-expressing cell population, ensuring experimental consistency and optimal protein yield during long-term cell culture.
20. Quantify secreted pACE2_1-740_-V5-His protein.
  a. Add 20 μL of V5-NanoTrap magnetic agarose slurry (ChromoTek) to a 1.5 mL reaction tube. The slurry has a binding capacity of 17.5 μg of recombinant V5-tagged protein (∼30 kDa) per 25 μL of beads.
  b. Wash the beads once with 200 μL of 1× PBS.
  c. Remove the supernatant by placing the tube in a magnetic stand.
  d. Incubate 500 or 250 μL of collected cell culture supernatant with 20 μL of V5-NanoTrap magnetic agarose slurry for 2 h at 4°C.
    ***Note:*** Use 500 μL of supernatant from non-transfected HEK293T cells as control.
  e. Wash magnetic V5 trap slurry with 500 μL 1× PBS containing 0.05% Tween.
  f. Transfer slurry in a new 1.5 mL reaction tube.
  g. Remove supernatant and add 10 μL of 2× Laemmli buffer (Sigma Aldrich).
  h. Heat for 3 min at 90°C.
  i. Separate proteins by SDS-PAGE, prepare in the same way and separate also a BSA (Thermo Fisher) for protein quantification diluted in Laemmli buffer. Typically use between 0.1 and 4.0 μg BSA per lane in a final volume of 10 μL.
  j. Stain the gel for 1 h in Coomassie staining solution with gentle agitation.
  k. Destain the gel in Coomassie destaining solution for 2–4 h with gentle agitation.
  l. Replace the destaining solution as needed when it becomes saturated with Coomassie dye to improve background clarity.
    ***Note:*** Place a crumpled tissue paper (e.g., paper towels) in the destain bath — it helps to absorb excess dye and accelerates background clearing.
  m. Remove destaining solution and add d. water to the gel.
  n. To quantify stained protein bands, capture an image of the stained gel on a white plate using a digital camera (e.g., SONY NEX5 E3.5/30 macro objective). The expected band of ACE2_1-740_-V5-His should appear at approximately 115 kDa. A typical Coomassie dye stained gel is presented in Figure 5.

### Preparation of a 96-well plate with HeLa cells expressing the RBD on the cell surface

**Timing: 3–4 days**

**Figure 6:**
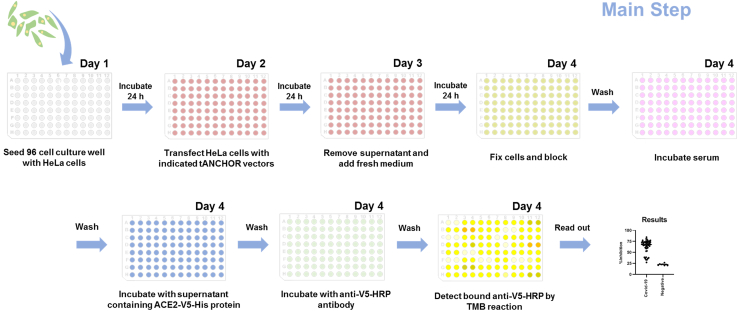
Key steps in a cell-based assay for evaluating the neutralizing activity of human serum against SARS-CoV-2 The workflow illustrates the protocol used when cells are transfected on Day 2 following seeding.

The following steps outline the preparation of a 96-well plate with HeLa cells expressing the RBD on the cell surface. Typically, this process takes 3 days but can be shortened to 1 day if cells are transfected on the day of seeding (Figure 6).

21. Trypsinize HeLa cells from a confluent 75 cm^2^ cell culture flask.
  a. Remove supernatant.
  b. Wash cells once with 10 mL sterile 1× PBS.
  c. Add enough pre-warmed (37°C) trypsin-EDTA to completely cover the cells.
  d. Place cell culture flask at 37°C for 3–6 min.
  e. Add 30 mL of pre-warmed (37°C) DMEM containing FBS.
    ***Note:*** Use a light microscope to observe cell detachment from the culture surface. Avoid prolonged trypsinization to maintain cell viability.
  f. Transfer cell suspension in a 50 mL cell culture tube.
  g. Centrifuge cells (900 rpm, 3 min, RT).
  h. Remove supernatant and wash cells by adding pre-warmed (37°C) 30 mL sterile 1× PBS.
  i. Centrifuge cells (900 rpm, 3 min, RT),
  j. Remove supernatant and add 20 mL pre-warmed (37°C) DMEM containing FBS.
22. Prepare HeLa cells for seeding in a 96-cell culture well.
  a. Count cells using an Improved Neubauer cell chamber.
  b. Dilute cell suspension with DMEM containing FBS to 1.5 × 10^4^ cells per 100 μL.
23. Add 100 μL cell suspension to each 96-well (TPP).
24. Incubate for 24 h at 37°C, 5% CO_2_, relative humidity 90%–95%.

***Note:*** Seeded HeLa cells should reach a maximum confluence of 70%–80% at the time of transfection. A higher cell density may reduce transfection efficiency. To accelerate the protocol, cells can be transfected 5 h after seeding, once they have settled. This approach shortens the protocol by one day. In this case, adjust the seeding cell density to 2.5 × 10^4^ cells per 100 μL.

25. Transfection of cells – use following instruction for upscaling:
  a. Calculate the total DNA required for transfection of HeLa cells. Use 0.3 μg of plasmid DNA per one 96-well, dilute plasmid DNA in 50 μL of serum- and antibiotic-free DMEM.
  b. Dilute separately for one 96-well 1 μL Metafectene (Biontex) in 50 μL of serum- and antibiotic-free DMEM.
  c. Mix by one-time pipetting and incubate for 20 min at RT, mix again by one-time pipetting.
26. Remove supernatant from cells.

**CRITICAL:** Do not allow the cells to dry in the following steps. Immediately add the transfection mix or medium after removing the supernatant.

27. Add 100 μL transfection mix to each well.
28. Incubate for 24 h at 37°C, 5% CO_2_, relative humidity 90%–95%.
29. After 24 h remove supernatant and add 100 μL medium containing 10% FBS.
30. Incubate for 24 h at 37°C, 5% CO_2_, relative humidity 90%–95%.
31. Verify HeLa cell transfection and cell adherence by examining mCherry fluorescence under a fluorescence microscope (e.g., Zeiss Axio Observer) and optional with a confocal laser microscope (e.g., Zeiss LSM780), see Figure 7 and for ref.^1^^,^^8^^,^^9^^,^^10^^,^^11^^,^^12^

**Figure 7:**
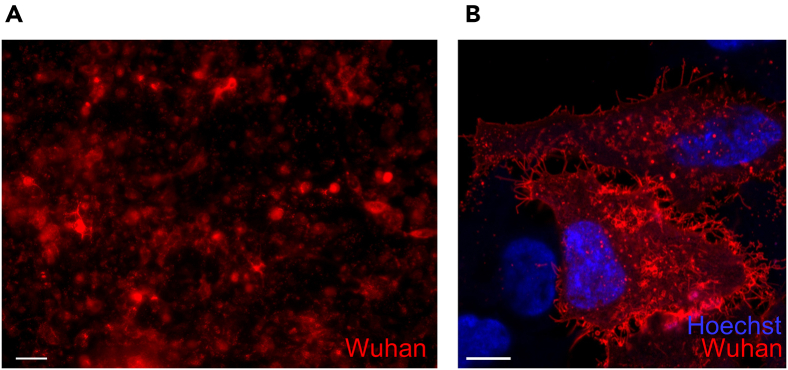
Subcellular localization of tANCHORed RBD in transfected HeLa cells (A) Epifluorescence image showing tANCHORed RBD fused to mCherry after fixation in a 96-well plate (Zeiss Axio Observer). (B) Confocal laser scanning microscopy (CLSM) image showing membrane-localized RBD expression (Zeiss LSM780). Cell nuclei are stained with Hoechst 33342. Scale bars: (A) 100 μm; (B) 10 μm.
32. Remove supernatant from cell layer.
33. Wash one-time with 300 μL of 1× PBS.
34. Add 100 μL of cell fixation buffer 2% PFA and incubate for 20 min at RT.

**CRITICAL:** Paraformaldehyde (PFA) is a known human carcinogen and irritant that affects the eyes, skin, and respiratory system. Always handle PFA powder in a certified chemical fume hood when preparing a 2% solution in 1× PBS. When working with the 2% PFA solution, ensure the room is well-ventilated with fresh air. Avoid inhalation of PFA fumes at all times. Follow local regulations and safety protocols when handling with PFA.

35. Remove PFA solution and wash two-time with 300 μL of 1× PBS.
36. Add 100 μL of blocking buffer and incubate for 1 h at RT.

***Note:*** Alternatively, cells can be stored in 1× PBS after PFA fixation for 3–4 days at 4°C. Seal the 96-well plate to prevent changes in salt concentrations due to cell drying.

### Testing of human serum on a prepared 96-well plate with displayed RBD on HeLa cells

**Timing: 6 h**

Human serum samples are tested on a 96-well plate containing fixed and blocked HeLa cells displaying the RBD on their surface. Serum binding is assessed by incubating the cells with diluted samples, followed by detection of ACE2_1-740_-V5-His binding inhibition. This allows quantification of neutralizing antibody activity against the displayed RBD.

37. Remove blocking buffer.
38. Add human test serum diluted in blocking buffer to each well and incubate for 1 h at RT.

***Note:*** We recommend starting the serum dilution at 1:20 in blocking buffer. The required amount of serum should be diluted in a deep well to facilitate pipetting from the 96 deep-well into the 96-well plate. This handling is necessary when comparing neutralizing activity against different SARS-CoV-2 variants.

39. Remove the diluted serum and wash once with 450 μL of washing buffer.
40. Add collected supernatant that contains ACE2_1-740_-V5-His protein. The amount should be 0.7 μg per well in a 96-well plate, applied in a total volume of 100 μL.
41. Incubate for 1.5 h at RT.
42. Wash three times with 450 μL of washing buffer.
43. Incubate for 45 min at RT 100 μL of 1:8,000 diluted detector antibody (mouse anti-V5-HRP, Thermo Fisher Scientific).
44. Wash five times with 450 μL of washing buffer.
45. Detect bound anti-V5-HRP.
  a. Add 80 μL of 3,3′,5,5′-tetramethylbenzidine (TMB) reaction solution.
  b. Incubate plate for 15–30 min at RT (protect plate from light) until a visible blue color develops.

***Alternatives:*** You can also use any TMB solution, such as the TMB Substrate Kit from Thermo Fisher.

46. Add 100 μL of 2 M H_2_SO_4_ to stop TMB reaction.
47. Read out the absorbance at 450 nm with a reference wavelength at 620 nm.
48. Calculate the neutralizing activity using the following Equation 1:

(Equation 1) $$\%\;\text{Neutralizing}\;\text{activity}=\left(1-\frac{A_{\text{sample}}}{A_{\text{total}}}\right)\times 100$$

A_sample_: Absorbance value at 450 nm – 620 nm when wells are incubated with human serum diluted in dilution buffer.

A_total_: Absorbance value at 450 nm – 620 nm when wells are not incubated with human serum, only with dilution buffer. This value represents the total binding of the ACE2_1-740_-V5-His protein to the displayed RBD.

## Expected outcomes

**Figure 8:**
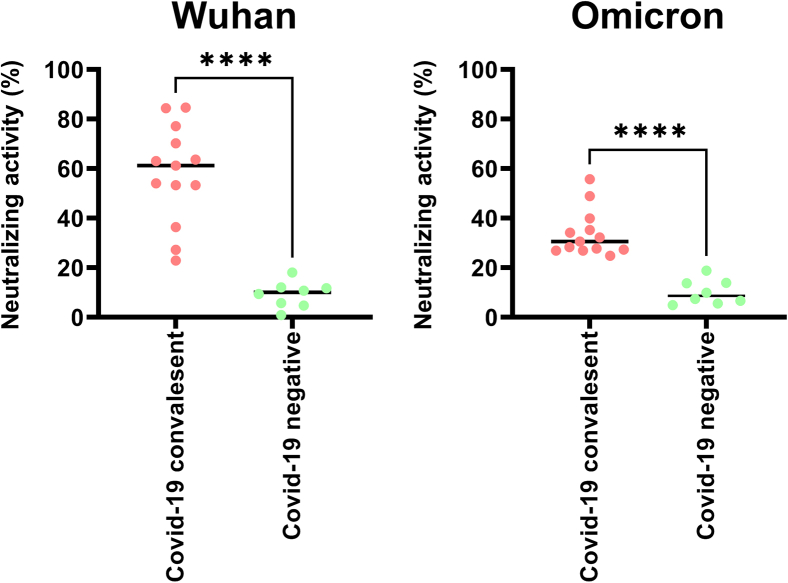
Representative results obtained from collected human serum samples Neutralization activity is assessed using HeLa cells expressing surface-displayed tANCHORed RBDs. Left panel: Neutralization measured against Wuhan-RBD. Right panel: Lower neutralization activity observed against Omicron-RBD. Groups are compared for significance by an unpaired two-tailed Student's t-test, ∗∗∗∗: *p* < 0.0001.

After performing the cell-based ELISA, the neutralization activity will reflect the percentage of inhibitory efficiency of the diluted serum from individuals. A typical example of results obtained from the COVID-19 convalescent and COVID-19 negative groups is shown in Figure 8.

## Quantification and statistical analysis

Samples should be measured in at least duplicates to achieve greater confidence in the results. This should be done as a form of control because high variance in the results from the two wells indicates a flaw in the experimental procedure. The mean can be plotted and compared with other SARS-CoV-2 individual groups, such as convalescent and vaccinated individuals. In this case, an unpaired two-tailed Student’s t-test is useful to detect statistical significance between two groups.

## Limitations

There are several limitations to the current protocol that warrant attention. One significant limitation is our focus on measuring the inhibition of the interaction between the RBD and ACE2. Our developed protocol for performing cell-based neutralization assays may not detect other potential neutralizing antibodies targeting the S2 domain or regions outside the RBD. Neutralizing antibodies against these regions could be relevant for estimating neutralization activity, particularly with emerging SARS-CoV-2 variants. Another critical consideration is the display of RBD on the cell surface, which could be influenced by emerging SARS-CoV-2 variants, potentially affecting its efficient presentation. Therefore, it is crucial to examine the localization of RBD on the cell surface using fluorescence microscopy for each new variant utilized in our developed method. We have investigated that slight variations in protein expression do not influence neutralization activity.^1^ However, for comparative reliability among different variants, transfection efficiency should be consistent. The mCherry tag can be used in this case for quantification by using the fluorescence readout. Finally, this protocol is limited to adherent cell lines, as suspended cells cannot be processed using an ELISA washer and would be lost during the washing steps.

## Troubleshooting

### Problem 1: Cells are detaching from the cell culture plate (related to step 31)

Under certain conditions, it can be observed after transfection and incubation that cells detach, and the HeLa cell layer is not homogeneous across all microplate wells.

### Potential solution

• Reduce plasmid DNA for transfection up to 0.2 μg per well.
• Optimize transfection protocol or use other transfection reagents like jetPRIME (Polyplus) where 0.1 μg/one 96-well plasmid DNA is sufficient.
• Shorten the incubation time with the transfection mixture.
• Transfect at lower cell confluency (e.g., 50%–60%) to reduce stress and improve viability.
• Use only well-maintained HeLa cells.
• Reduce the number of cells per well.
• Use plasmid isolation kits yielding low endotoxin in DNA preparations.
• Use freshly prepared paraformaldehyde (PFA) for fixation.
• Extend PFA fixation time to 30–40 min for improved cell morphology and protein preservation.

### Problem 2: Cells are detaching from the cell culture plate during washing steps (related to steps 33, 35, 39, 42, and 44)

HeLa cells are typically adherent and resistant to detachment during washing steps. We employed an ELISA washer with an adjustable flow rate. We used an ELISA washer BioTec 405 (Agilent Technologies) with a flow rate of 3.^1^^,^^4^ If cell detachment occurs during washing steps, optimize washing conditions.

### Potential solution

**Figure 9:**
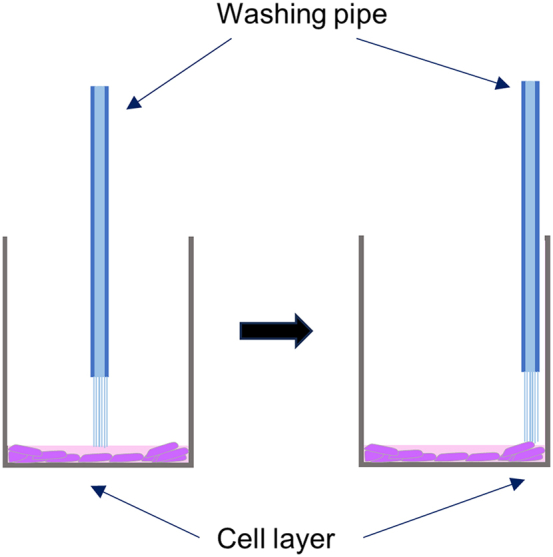
Improving cell layer stability during washing steps The cell layer is more stable when cells are attached to the well wall. Position the washing pipe as close as possible to the wall of the 96-well plate.
• If a low flow rate is not achievable, wash by hand pipetting.

• optimize the flow rate of the wash buffer.
• Position the flow pipe close to the wall of the 96-well plate and minimize the flow rate (Figure 9).

### Problem 3: Low absorbance value for the total binding of ACE2_1-740_-V5-His to the displayed RBD (related to step 48)

High absorbance values are necessary to ensure precision in monitoring neutralizing activity. A low value compromises assay sensitivity. The optimal absorbance (450 nm – 620 nm) should range between 0.7 and 1.0.

### Potential solution

• Check for transfection efficiency.
• Optimize transfection method.
• Try using alternative transfection reagents, such as jetPRIME, which may be more effective for cell lines that are difficult to transfect.
• Check for cell detachment and address as described above.

### Problem 4: High background on controls without ACE2_1-740_-V5-His incubation (related to step 48)

It is necessary to check how strongly the anti-V5-HRP antibody causes background. Under our our blocking conditions we observed low background levels, ranging from 0.1 to a maximum of 0.2 absorbance value. If a different detector antibody is used, follow these strategies to minimize nonspecific background.

### Potential solution

• Increase Tween 20 concentration up to 0.1% in the wash buffer.
• Optimize washing steps and increase the hold time during washes.
• Prevent cells from drying out, because this will increase unspecific antibody binding.
• Try using a different anti-V5-HRP as detector antibody for the V5 tag.
• Use higher dilution of the anti-V5-HRP detector antibody.
• Try using a different blocking buffer in order to reduce unspecific binding with serum from the species in which the secondary antibody was raised, similar to previously described methods.^13^^,^^14^

### Problem 5: Unrelated antibodies targeting RBD (related to step 38)

If specific neutralizing antibodies against a new, emerging SARS-CoV-2 variant need to be measured, antibodies targeting the RBD induced by a previous variant or vaccination against the spike protein will also be monitored. Discriminating between antibodies targeting new versus old RBDs is challenging due to sequence similarities.

### Potential solution

• Preincubate the diluted human serum on HeLa cells expressing a former SARS-CoV-2 RBD variant, then test the preabsorbed serum on cells displaying the new, emerging SARS-CoV-2 RBD variant. This procedure for enriching specific antibodies from a pool of antibodies was previously described.^8^

## Resource availability

### Lead contact

Further information and requests for resources and reagents should be directed to and will be fulfilled by the lead contact, Daniel Ivanusic (daniel.ivanusic@web.de).

### Technical contact

Further information and requests for resources and reagents should be directed to and will be fulfilled by the technical contact, Daniel Ivanusic (daniel.ivanusic@web.de).

### Materials availability

Plasmids containing the patented tANCHOR technology generated in this study will be made available upon request, but payment and a completed materials transfer agreement may be required.

### Data and code availability

This study did not generate or analyze datasets or codes.

## Acknowledgments

We gratefully thank the 10.13039/100021130Federal Ministry for Economic Affairs and Climate Action of Germany for funding this project (funding code: 16KN074245). We thank all data contributors, i.e., the authors and their originating laboratories responsible for obtaining the specimens, and their submitting laboratories for generating the genetic sequence and metadata and sharing via the GISAID Initiative, on which the SARS-CoV-2 RBD sequences are based.

## Author contributions

D.I. invented the assay concept and prepared figures. D.I. and N.B. wrote the manuscript.

## Declaration of interests

The non-profit organization Peter und Traudl Engelhorn Foundation holds a patent application for the tANCHOR system, where D.I. is listed as an inventor.
